# Improved detection of methanogenic archaea by validating 16S rRNA gene primers using defined archaeal–bacterial mock communities

**DOI:** 10.1093/ismeco/ycag295

**Published:** 2026-10-13

**Authors:** Kristel Mijnendonckx, Ann Provoost, Carla Smolders, Natalie Leys

**Affiliations:** Microbial Biotechnology Unit, Belgian Nuclear Research Centre, SCK CEN, 2400 Mol, Belgium; Microbial Biotechnology Unit, Belgian Nuclear Research Centre, SCK CEN, 2400 Mol, Belgium; Microbial Biotechnology Unit, Belgian Nuclear Research Centre, SCK CEN, 2400 Mol, Belgium; Microbial Biotechnology Unit, Belgian Nuclear Research Centre, SCK CEN, 2400 Mol, Belgium

**Keywords:** archaea, 16S rRNA gene sequencing, mock community

## Abstract

Archaea play essential roles in global biogeochemical cycles, yet their diversity in environmental samples remains understudied. Limited coverage of universal primers toward archaea, their very low abundance relative to bacteria in the studied samples, inefficient cell lysis, and the incompleteness of reference databases complicate archaeal 16S rRNA gene sequencing. Furthermore, standardization lacks due to the absence of commercially available mock communities. Here, we developed a mock community composed of mixtures of DNA from six archaeal and four bacterial strains with known genome sizes and 16S rRNA gene copy numbers. Five mock compositions with different archaeal–bacterial abundance ratios were used to evaluate two universal primer pairs and eight archaea-specific methods. The universal primer pairs detected all taxa but consistently overestimated bacterial abundance, indicating to be less suitable for environments where archaea are more dominant. Archaeal-specific primer pairs showed variable specificity, with some exhibiting substantial bacterial co-amplification under certain conditions. The Arch349F–A806R primer pair demonstrated the highest archaeal specificity, the least technical variability, the most consistent agreement with expected mock compositions, and minimal artefactual amplification when applied to a complex bacterial mock community. To show its applicability on environmental samples, this method was applied on Boom Clay pore water samples, which indicated a much higher archaeal diversity than previously shown with a standard commercial method. Overall, this study showed the necessity of primer validation through mock communities and identified Arch349F–A806R as a promising primer pair for archaeal 16S rRNA gene sequencing, especially in samples containing methanogenic archaea.

## Graphical Abstract

**Graphical Abstract:**
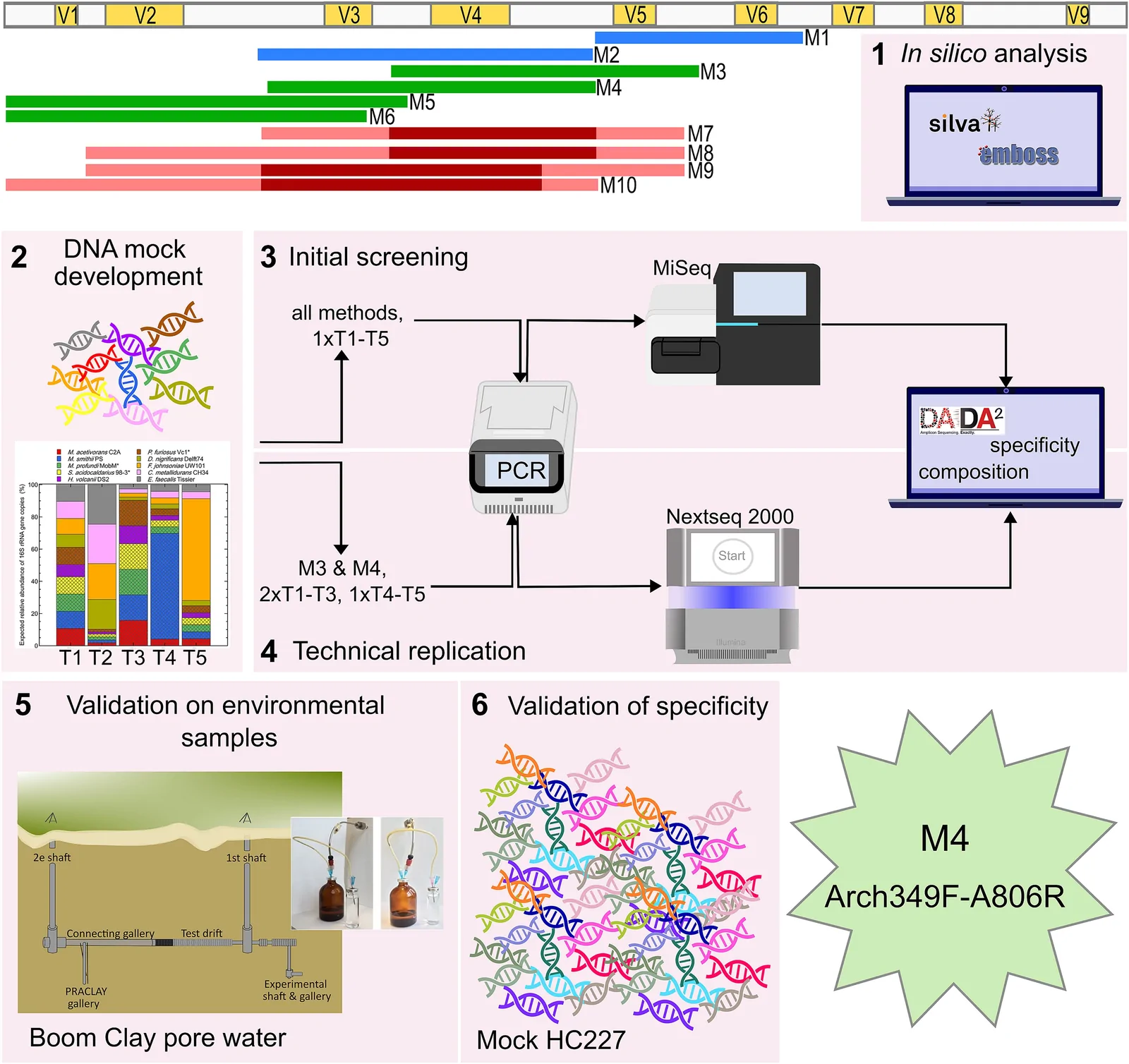

## Introduction

Microbial community dynamics have been extensively studied in a variety of environments, with most research focusing primarily on bacteria [1–4]. However, archaea—despite being a diverse and ecologically significant domain of microbial life—remain comparatively understudied. Archaea are found in a variety of extreme and non-extreme environments, including marine sediments, hydrothermal vents, and the human microbiome [5, 6]. Recent advancement in high-throughput sequencing technologies and computational methods have led to the discovery of numerous novel archaeal lineages [5]. This expanded not only the view on the diversity of Archaea, but it also strengthened the understanding of their metabolic and ecological role. Archaea play a crucial role in geochemical cycles, e.g. the carbon and nitrogen cycle through processes such as methanogenesis and anaerobic methane oxidation which are exclusively carried out by archaea [7].

To date, 16S rRNA gene sequencing remains the most widely used approach to investigate microbial community composition and dynamics, despite its limitations [8–12]. Archaeal research is further complicated by the inefficient lysis methods that hinder DNA extraction from archaeal cells [13–15], the limited coverage of universal primers toward archaea [16–20], their very low abundance compared to other organisms in the studied samples [13, 19], and by the incompleteness of reference databases that limit accurate taxonomic classification [19]. Consequently, a multitude of archaeal-specific primers sets targeting different regions of the 16S rRNA gene have been developed [15, 20–24]. While few studies used internal references in their wet-lab experiments, most of the archaea-specific primer pairs are selected based on *in silico* analysis lacking further experimental validation with necessary controls such as mock communities [16, 22, 25–28]. This lack of standardization reduces confidence in the comparability and reproducibility of archaeal community profiling studies. Although several commercially available mock communities are currently available for standardizing 16S rRNA gene sequencing studies, most are focused on bacterial research and typically lack archaea, except for a few that include a single archaeal strain [29–32]. Consequently, there is a clear need for experimentally validated mock communities that include multiple archaeal taxa, to enable thorough assessment of primer performance and methodological biases.

We aimed to develop and validate a method for accurately identifying and quantifying archaea in environmental microbial communities, with a special focus on methanogenic archaea. To this end, we created a mock community comprising DNA from six archaeal and four bacterial species in various ratios. We included three distinct methanogenic archaea and three nonmethanogenic model organisms, spanning five different orders and multiple major ecological/physiological types. Furthermore, the mock community was complemented with DNA from four bacteria either co-occurring with methanogens or posing methodological challenges such as nonspecific amplification or lysis resistance. We then evaluated the performance of 10 primer pairs targeting diverse regions of the 16S rRNA gene for their ability to assess archaea in mixed communities.

## Materials and methods

### Primers

An overview of the different methods and the detailed primer combinations and Polymerase Chain Reaction (PCR) amplification conditions used in this study is presented in Table 1. Primers contained an Illumina adapter overhang sequence: 5′ – TCGTCGGCAGCGTCAGATGTGTATAAGAGACAG – 3′ for the forward primers and 5′ – GTCTCGTGGGCTCGGAGATGTGTATAAGAGACAG – 3′ for the reverse primers.

**Table 1:** Overview of the different primer pairs and annealing temperatures used in this study.

Method	Primer name	Sequence 5′-3′	Target region	Annealing temp	Amplicon length ^a^ (bp)	Ref.
M1	807F 1050R	GGATTAGATACCCBRGTAGTC AGYTGDCGACRRCCRTGCA	V5-V6	60°C^b^	244	[57]
M2	Pro341F Pro805R	CCTACGGGNBGCASCAG GACTACNVGGGTATCTAATCC	V3-V4	56°C	464	[58]
M3	U519F Ar9R	CAGCMGCCGCGGTAA CCCGCCAATTCCTTTAAGTTTC	V4-V5	71°C	408	[56, 68]
M4	Arch349F A806R	GYGCASCAGKCGMGAAW GGACTACVSGGGTATCTAAT	V3-V4	64°C	459	[23, 56]
M5	SSU1bF^*^ SSU520R	CCGGTTGATCCWGSCRBA GCTACGRRYGYTTTARRC	V1-V3	58°C	520	[20]
M6	SSU1bF^*^ SSU468R	CCGGTTGATCCWGSCRBA GNDCNGCNGGTDTTACCG	V1-V2	58°C	468	[20]
M7	ARC344f ARC915r U519F A806R	ACGGGGCGCAGCAGGCGCGA GTGCTCCCCCGCCAATTCCT CAGCMGCCGCGGTAA GGACTACVSGGGTATCTAAT	V4	64°C 60°C	571 288	[69] [56]
M8	A109f ARC915r U519F A806R	ACKGCTCAGTAACACGT GTGCTCCCCCGCCAATTCCT CAGCMGCCGCGGTAA GGACTACVSGGGTATCTAAT	V4	50°C 60°C	806 288	[69, 70] [56]
M9	A109f ARC915r ARC344f ARC744r	ACKGCTCAGTAACACGT GTGCTCCCCCGCCAATTCCT ACGGGGCGCAGCAGGCGCGA CCSGGGTATCTAATCC	V3-V4	50°C 50°C	806 400	[69, 70] [69, 71]
M10	SSU1bF^*^ A806R ARC344f ARC744r	CCGGTTGATCCWGSCRBA GACTACVSGGGTATCTAAT ACGGGGCGCAGCAGGCGCGA CCSGGGTATCTAATCC	V3-V4	58°C 50°C	806 400	[20, 56] [69, 71]

^a^Amplicon length presented here is based on *Escherichia coli.* Exact values can slightly vary among different strains. The length of the Illumina adapter is not included.

^b^A touchdown PCR was performed starting at 60°C with a decrease of 2°C every six cycles.

^^*^^This primer was modified from [20] TCCGGTTGATCCYGCBRG to enable amplification of *M. smithii.*

### *In silico* primer evaluation

The performance of the primer pairs was evaluated *in silico* using SILVA TestPrime 1.0 on the nonredundant SILVA Reference database (release SSURef 138.2 NR) containing 510 495 sequences [21]. We compared coverages allowing up to a maximum of two mismatches as this is assumed to be tolerated in a standard PCR reaction [33]. SILVA TestPrime 1.0 did not allow evaluation of a nested PCR approach, so instead the ‘primersearch’ command of EMBOSS (version 6.6.0.0) was used to estimate coverages for these nested primer pairs [34]. To estimate coverages instead of raw hits, we used the following approach: (i) The SSURef 138.2 NR database was separated in archaeal and bacterial FASTA files to enable independent estimation of coverages for both domains. (ii) Eligible sequences were identified by a primer search against the databases prepared in step (i) with lenient conditions (i.e. five mismatches) followed by a filtering based on primer binding positions (e.g. for primer SSU1bF, sequences with a binding site located beyond the first 100 bases were removed). The outcome of this analysis was used as eligible sequences. (iii) We simulated the first PCR by applying a primer search on the SSURef 138.2 NR databases prepared in step (i) with the outer primer pairs allowing up to a maximum of two mismatches, followed by a filtering based on the expected amplicon length. (iv) The nested PCR reaction was evaluated on the output of step (iii) with two allowed mismatches. Finally, primer coverage was calculated as the proportion of sequences retained after each step relative to the total number of eligible sequences. SILVA TestPrime 1.0 was also applied on the outer primer pair of the nested approaches to compare both methods. In addition, ‘primersearch’ on the SSURef 138.2 NR databases prepared in step (i) was used to evaluate the primer pairs which were too degenerative for SILVA TestPrime 1.0.

### Development of the DNA mock

We developed a DNA mock community comprised of mixtures of DNA from 10 strains including six archaea and four bacteria (Table 2, Supplementary Table 1). Our focus was mainly on methanogenic archaea so we selected three distinct methanogenic archaea, including *Methanosarcina acetivorans* C2A, a model organism for studying aceticlastic and reverse methanogenesis [35], *Methanobrevibacter smithii* PS, a model organism for studying methanogenesis in the human gut [36], and *Methanolobus profundi* MobM, a methylothrophic methanogen isolated from deep subsurface sediments [37]. In addition, we included three nonmethanogenic model organisms, namely *Sulfolobus acidocaldarius* 98-3 [38], *Haloferax volcanii* DS2 [39], and *Pyrococcus furiosus* Vc1 [40]. The mock community also included DNA from four bacteria. *Flavobacterium johnsoniae* subsp. *Johnsoniae* UW101 and *Desulfotomaculum nigrificans* Delft 74 were added as representatives of bacteria previously found in Boom Clay pore water samples together with methanogenic archaea (Supplementary Fig. 1). *Cupriavidus metallidurans* CH34 was added as it often amplifies with archaea-specific primers [41]. Finally, *Enterococcus faecalis* Tissier was included as a bacterium that is difficult to lyse, for constructing cell mock communities enabling the comparison of DNA extraction methods [42]. We created five distinct mock communities, each having a different amount of 16S rRNA gene copies of each strain (Fig. 1). DNA from *M. acetivorans* C2A (DSM 2834), *M. smithii* PS (DSM 861), *M. profundi* MobM (DSM 21 213), *P. furiosus* Vc1 (DSM 3638), *D. nigrificans* Delft 74 (DSM 574), and *F. johnsoniae* subsp. *Johnsoniae* UW101 (DSM 2064) was obtained from the DSMZ-German Collection of Microorganisms and Cell Cultures GmbH. *Cupriavidus metallidurans* CH34 (DSM 2839), *H. volcanii* DS2 (DSM 3757), and *E. faecalis* Tissier (DSM 20 478) were obtained from our in-house culture collection, while *S. acidocaldarius* 98-3 (DSM 639) was kindly provided by Prof. Dr. ir. Eveline Peeters (VUB, Brussels, Belgium). These four strains were cultivated to late exponential phase, and genomic DNA was extracted using the QIAamp DNA mini kit (Qiagen, The Netherlands) following the manufacturer’s protocol. To this end, *S. acidocaldarius* 98-3 was grown in basal Brock medium supplemented with 2 g l^−1^ sucrose, 1 g l^−1^ NZ-amine, and 20 μg ml^−1^ uracil at 75°C [43]. *Haloferax volcanii* DS2 was cultivated in DSMZ 97 medium at 37°C [44]. *Cupriavidus metallidurans* CH34 was cultured in Tris-buffered mineral medium supplemented with 0.2% (wt/vol) gluconate (MM284) [45] at 30°C. Finally, *E. faecalis* Tissier was grown in Lysogeny Broth (LB; Thermo Fisher Scientific, Belgium) at 37°C. For genomic DNA extracted in-house, purity was assessed by NanoDrop spectrophotometry (Thermo Fisher Scientific, Belgium), and integrity was evaluated by agarose gel electrophoresis. DNA obtained from DSMZ was used as supplied. No dedicated species-specific contamination assessment was performed for individual DNA stocks before preparation of the mock communities.

**Table 2:** Microbial strains present in the mock community.

Strain	DSM number	Genome size (bp)	GC content (%)	16S rRNA gene copies	Nr. of 16S rRNA gene variants
*M. acetivorans* C2A	2834	5 751 492	43.5	3	2
*M. smithii* PS	861	1 853 160	31	2	2
*M. profundi* MobM	21 213	3 107 200	42.5	1	n.a.
*S. acidocaldarius* 98-3	639	2 225 959	36.7	1	n.a.
*H. volcanii* DS2	3757	4 012 900	65	2	1
*P. furiosus* Vc1	3638	1 908 256	40.8	1	n.a.
*D. nigrificans* Delft 74	574	3 052 187	46.3	6	6
*F. johnsoniae* subsp*. Johnsoniae* UW101	2064	6 096 872	34.1	6	4
*C. metallidurans* CH34	2839	6 913 352	63.5	4	1
*E. faecalis* Tissier	20 478	2 866 949	37.4	4	3

n.a., not applicable.

**Figure 1:**
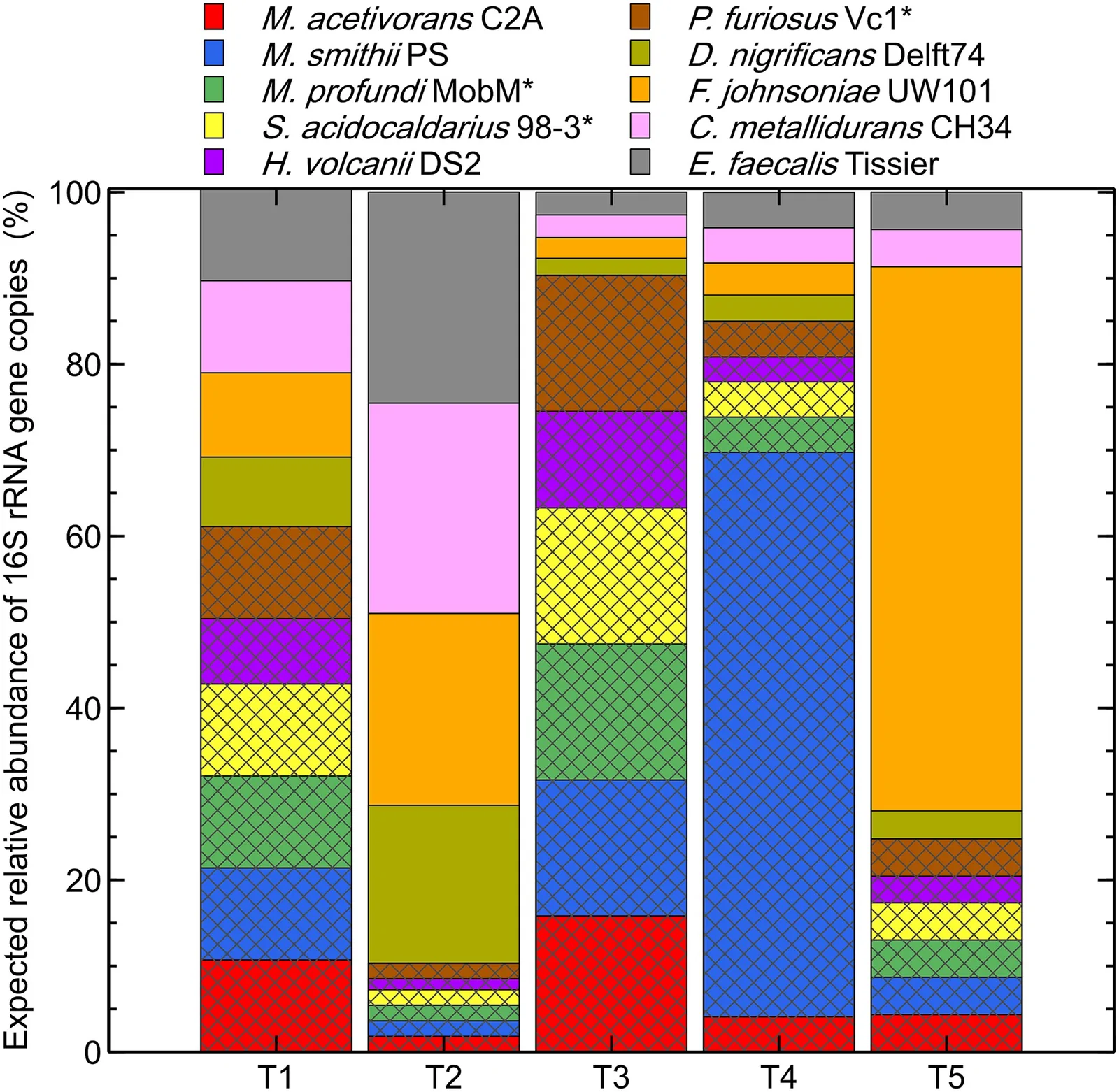
Bar plot visualizing the expected relative abundances of 16S rRNA gene copies determined based on genome size, 16S rRNA gene copy number, and the input DNA mass per strain. Mock community T1 contained an approximately equal distribution of 16S rRNA gene copies of each strain. T2 had a higher relative abundance of bacterial 16S rRNA gene copies, while T3 contained archaeal 16S rRNA gene copies in a higher relative abundance. In T4 and T5, 16S rRNA gene copies of one strain were present at a disproportionately higher abundance compared to the others. Archaea are indicated with a diagonal cross. Strains that contain a single copy of the 16S rRNA gene are indicated with an ^*^.

DNA obtained from each strain separately was mixed in varying ratios to create five distinct mock communities, each containing a total of 300 ng DNA (Fig. 1). The amount of genomic DNA added per strain was calculated such that the expected number of 16S rRNA gene copies from each strain was equal to the target relative abundance, accounting for both genome size and 16S rRNA gene copy number. Mock community T1 contained an approximately equal distribution of 16S rRNA gene copies of each strain. T2 had a higher relative abundance of bacterial 16S rRNA gene copies, while T3 contained archaeal 16S rRNA gene copies in a higher relative abundance. In T4 and T5, 16S rRNA gene copies of one strain were present at a disproportionately higher abundance compared to the others. Overall, T1, T3, and T4 were dominated by archaeal 16S rRNA gene copies, while T2 and T5 were dominated by bacterial 16S rRNA gene copies (Fig. 1).

### PCR conditions

Thirty nanograms of DNA were used as template and were amplified using Phusion High-Fidelity Polymerase (Thermo Fisher Scientific, Belgium). PCR conditions were similar for all methods, i.e. initial denaturation for 2 min at 98°C, 30 cycles of 10 s at 98°C followed by 30 s at a specific annealing temperature and 1 min at 72°C, and a final elongation of 10 min at 72°C. For the nested PCR reactions, the number of cycles was decreased to 25. Annealing temperatures (Table 1) were selected based on the original publications and were first tested on the DNA of all strains separately. When gel electrophoresis indicated that the PCR reaction was not successful for one of the individual strains, annealing temperatures were optimized performing a gradient PCR, first on 30 ng DNA of *S. acidocaldarius* and *E. faecalis* as template. The optimal conditions were then confirmed on all strains before continuing to the amplification of the different mock communities. All PCR products were purified with the Wizard® SV Gel and PCR CleanUp System (Promega, The Netherlands) according to the manufacturer’s protocol.

### Boom Clay pore water sampling and analysis

The Boom Clay formation was accessed through the HADES Underground Research Facility (URF) of SCK CEN (Mol, Belgium), a concrete-lined gallery at a depth of 225 m below the surface. Boom Clay pore water is classically sampled from the HADES URF by means of porous filter screens inside piezometers [46, 47]. The piezometers sampled in this study were located in the PRACLAY gallery (13 samples), the connecting gallery (2 samples), and the test drift (2 samples) of the HADES URF (Supplementary Fig. 2). All piezometers were made of stainless steel, and the stainless steel porous filter screens had an average pore diameter of 9 μm [47]. Water samples were taken in anoxic conditions and transferred to an anaerobic glove box (99% Ar/1% H_2_) immediately after sampling. Fifty ml of pore water was filtered (0.22 μm Supor® PES, Pall Corporation, Belgium) to collect all microorganisms. DNA was extracted according to the procedure described in [48]. Method M4 (Table 1) was used to amplify two Boom Clay pore water samples (Sample 15 and 17).

### Mock HC227

Method M4 (Table 1) was used to amplify—in duplicate—HC227, a complex bacterial mock community consisting of DNA from 227 strains. Strains belong to eight phyla (Actinobacteria, Bacteroidetes, Deinococcus-Thermus, Firmicutes, Fusobacteria, Planctomycetes, Proteobacteria, and Verrucomicrobia), 18 classes, 47 orders, 85 families, 175 genera, and 197 species [49].

### Sequencing and reads processing

In a first screening experiments, one replicate of all samples was generated. These sample amplicons were sequenced on the Illumina MiSeq platform at BaseClear B.V (the Netherlands) according to the manufacturer guidelines. To assess technical variation across repeated experiments, two additional replicates for mock T1, T2, and T3 and one additional replicate for mock T4 and T5 were generated with M3 and M4 using exactly the same mock communities and amplification conditions as in the first experiment. Samples were sequenced on the NextSeq 2000 platform at BaseClear B.V (the Netherlands) according to the manufacturer guidelines. In addition, purified DNA of all 17 Boom Clay pore water samples was sent to BaseClear B.V (the Netherlands) for high-throughput amplicon sequencing of the V3–V4 hypervariable region of the 16S rRNA gene for bacteria and the V4–V5 hypervariable region of the 16S rRNA gene for archaea on the Illumina MiSeq platform according to the manufacturer guidelines. Primers were removed from the 16S rRNA gene sequencing data using cutadapt [50]. Afterwards, reads were processed according to the DADA2 pipeline [51], with processing done separately for each primer pair. Reads with ambiguous, poor-quality bases and more than two expected errors were discarded. Filter and trim parameters different from the default settings are specified in Supplementary Table 2. The paired reads were merged, chimeras were identified and removed. Only amplicon sequence variants (ASVs) with more than two reads in two different samples were retained. For the analysis of Boom Clay pore water samples with M4, this filtering step was not included. Taxonomy was assigned to the ASVs using the naive Bayesian classifier method implemented in DADA2 [51] with the Silva taxonomic training dataset (version 138.2) as a reference. For the analysis of the Boom Clay pore water and Mock HC227 samples, the Silva taxonomic training dataset (version 138.2) was combined with the SBDI Sativa curated 16S GTDB database [52] to increase the number of archaea sequences. As such, 3435 additional unique archaeal 16S rRNA gene sequences were added to the reference database.

The 16S rRNA amplicon sequencing data were further analyzed in R version 4.5.2 with the R package phyloseq (version 1.540.) [53]. The R package chkMocks (version 0.1.04) was used to calculate Spearman correlation coefficients between each sample and the theoretical composition [54]. The R package RCM (version 1.26.0) was used as ordination method to visualize the composition obtained in each condition compared the theoretically expected composition [55]. This exploratory ordination method was chosen as it uses ASV counts instead of frequencies, is not prone to rarefaction, and can handle overdispersion and zero counts [55]. Finally, the distance to the theoretical composition was calculated based on robust Aitchison distances using the R packages vegan (version 2.7-2). To account for bias attributable to nonspecific bacterial amplification in the archaeal-specific methods, we applied a penalty term into the overall performance score for each method. Specifically, the final score was calculated as the sum of the Aitchison distance to the theoretical composition and the fraction of bacterial sequences multiplied by a penalty parameter. As nonspecific amplification of bacteria is a major disadvantage in archaea-targeted 16S rRNA gene sequencing, we set the penalty at 10. This choice strongly penalizes methods with substantial bacterial contamination while retaining sensitivity to compositional accuracy. The sequence datasets generated and analyzed during this study are available in the NCBI Sequence Read Archive (SRA) repository (PRJNA1444866).

### Statistical analysis

To test whether archaea-specific methods differed in performance across the five mock communities, a Friedman rank-sum test was performed on the penalized Aitchison distance, with mock community as the blocking factor. Pairwise post hoc comparisons were performed with the Nemenyi’s all-pairs test using the R package PMCMRplus (version 1.9.12). As a complementary targeted analysis, M4 was compared with every archaea-specific method. Within each mock community, paired differences in penalized Aitchison distance score were calculated as comparator score minus M4 score, such that positive values favored M4. Afterwards, an exact blocked permutation test was applied by enumerating all 2^5^ = 32 possible sign-flip permutations of the five paired mock-level differences. The *P*-value was calculated as the proportion of permuted mean differences at least as extreme as the observed difference followed by Holm adjustment. Furthermore, a nonparametric block bootstrap with 10 000 resamples of mock community as the resampling unit was used to obtain descriptive 95% percentile confidence intervals for the mean paired difference.

## Results and discussion

### *In silico* primer evaluation

We selected 10 different primer pair combinations, including two universal methods (M1 and M2) [21] and eight archaea-specific methods (M3–M10) (Table 1). The latter comprised a primer pair from a commercial approach (M3) [23, 56], a primer pair developed for quantitative PCR using fluorogenic probes (M4) [20], two more recent methods (M5 and M6) [20], and four nested PCR (M7–M10) approaches as these are often used to increase archaeal specificity [57, 58]. For one of the nested methods (M8 and M9), we used a distinct second primer to explore its impact. Due to the rapid discovery of new archaeal lineages, previously validated primers may no longer provide optimal coverage of current diversity. Therefore, we first evaluated the primer pairs *in silico* (Supplementary Table 3). In general, *in silico* analysis confirmed that both universal primer pairs (M1 and M2) have a high coverage for both Archaea and Bacteria (Supplementary Table 3). However, they showed low specificity for Archaea, which can lead to misinterpretations when evaluating relative abundances in mixed microbial communities. The primer pairs used in M3 and M4 showed a relatively high coverage for Archaea even without any mismatch (78.4% and 71.6%, respectively), which further increased up to 89.8% and 89.5% (one mismatch) and 92.5% and 94.7% (two mismatches), respectively (Supplementary Table 3). The primer pair Arch349F–A806R (M4) seemed specific for Archaea as Bacterial coverage is only 1% when two mismatches are allowed (Supplementary Table 3). In contrast, the specificity of primer pair U519F–Ar9R (M3) decreased with two mismatches, and its coverage of the Bacterial domain increased to 47.6% (Supplementary Table 3). Although Bahram *et al.* [20] performed an *in silico* analysis for primer pairs SSU1bF–SSU520R and SSU1bF–SSU468R with Silva TestPrime, we could not reproduce the data due to excessive degeneracy. Nevertheless, we included these primer pairs in our study as SSU1bF–SSU520R was recommended for short amplicons and SSU1bF–SSU468R showed a relatively high coverage for archaea (70.1%). However, as PCR amplification of *M. smithii* PS DNA failed consistently, SSU1bF was altered (SSU1bF^*^) in order to enable DNA amplification of *M. smithii* PS and all other strains, so we used this primer instead of the original SSU1bF. We estimated coverages with the ‘primersearch’ command of EMBOSS as primers were too degenerative for Silva TestPrime. ‘Primersearch’ indicated high specificity for both methods and good coverage if mismatched were allowed (Supplementary Table 3). The ‘primersearch’ command of EMBOSS was also used to calculate coverages for the nested approaches as Silva TestPrime does not allow this analysis. The primer pairs used for the nested approaches ARC344f–ARC915r (M8), A109f–ARC915r (M8 and M9), and SSU1bF^*^–A806R (M10) were all specific for Archaea. However, allowing mismatches was necessary to improve their coverage. Based on *in silico* analysis, it is not expected that the nested approach would decrease the coverage, although a higher decrease in coverage was observed with primer Arc344f–Arc744r (M9) (Supplementary Table 3). Notably, *in silico* analysis indicated none of the primer pairs were able to target Crenarchaeota or Euryarchaeota with the tested settings (Supplementary Fig. 3).

### Primer evaluation using the developed mock communities

All primer pairs were first evaluated using DNA from each individual strains, with each strain tested separately for PCR amplification under the selected conditions before amplifying the different mock communities. All primer pairs (M1–M10) were able to amplify DNA from all individual strains, including *S. acidocaldarius* 98-3 and *M. smithii* PS, belonging to the Crenarchaeota and Euryarchaeota, respectively (data not shown). This discrepancy between *in silico* and *in vitro* analysis emphasizes the necessity of wet-lab validation to accurately assess primer performance. Although all individual archaeal DNAs were PCR amplifiable with M6, no amplification was observed for the mock communities (data not shown). Putatively, the incorporation of multiple N and D as degenerate bases increases the number of possible primer variants, lowering the effective concentration of perfectly matching primer sequences and resulting in a reduced amplification efficiency in mixed communities [59]. Consequently, this primer pair was excluded from further use. All other primer pairs (M1–M10, except M6) were able to produce amplicons with the different mock communities and were analyzed in more detail.

#### Reads processing

The total number of reads obtained after sequencing the amplicons generated by the nine different primer pairs (M1–M10, excluding M6) across the different mock communities (T1–T5) ranged from 12 341 reads for M2 on mock T2 to 63 726 reads for M7 on mock T3 (Supplementary Fig. 4). In general, methods with the shortest amplicon length, i.e. M1, M7, and M8 resulted in the highest amount of reads, and the amount of reads decreased with increasing amplicon length, except for M5 which resulted in more reads than M2 although the amplicon length was larger.

Further processing of the reads indicated that M5 resulted in the highest read loss and only 47.40% ± 3.63 of the reads were retained after filtering (Supplementary Fig. 5). This amount further decreased until 37.40% ± 4.89 of the initial reads after removing chimera. This high loss might be due to the amplicon length, which is at the limit of the 2 × 300 bp sequencing approach. Due to the rapid decrease of the reverse read toward the 3′ end of the read, several reads were lost after filtering. On the other hand, this method was the least prone to chimera. M1 retained most of the reads after filtering (92.44% ± 1.22), but this method seemed most prone to chimera as 27.4% ± 3.1 of the reads were lost after chimera removal and the total loss of reads was similar as most other methods. The nested approaches (M7–M10) were also prone to chimera, but this can be expected due to the two PCR reactions that are carried out in these method [60]. Finally, M3–M5 were quite comparable with respect to the amount of reads retained. These observations are consistent with previously published studies, which have demonstrated that primer selection, amplicon length, and other methodological parameters can influence the outcomes of 16S rRNA gene sequencing [21, 26, 61]. Nevertheless, the number of reads remaining after data processing was sufficient to ensure adequate sequencing depth and coverage for downstream analyses.

#### Specificity of the primers

Both universal methods (M1 and M2) successfully detected all strains present in the mock communities, indicating that both approaches can simultaneously detect bacteria and archaea (Fig. 2). However, both methods M1 and M2 consistently overestimated the bacterial abundance. This was also reflected in the very low Spearman correlation coefficients between the experimental and theoretical community compositions (Fig. 2). Only in the mock community T2, where the four bacteria are highly dominant, a high Spearman correlation was observed. In addition, the relative abundances of most bacterial strains were comparable with the expected composition in T5, except for *E. faecalis*, which was overrepresented (Fig. 2, Supplementary Fig. 6). For the archaea taxa, the abundances of *H. volcanii* and *S. acidocaldarius* were estimated reasonably well across most conditions, particularly by M2. In contrast, *M. profundi* was almost consistently overestimated (Fig. 2, Supplementary Fig. 6). Taken together, these results suggest that universal methods—with a slight preference for M1—may be suitable for environments where bacteria are highly dominant over archaea. This is in agreement with the study of Takahashi *et al.* [58] reporting results for communities where archaea only represented 2% of the total population (determined with q-PCR). However, our results show that the universal methods M1 and M2 are not suitable to monitor archaea abundance in environments expected to be dominated with archaea [62–64].

**Figure 2:**
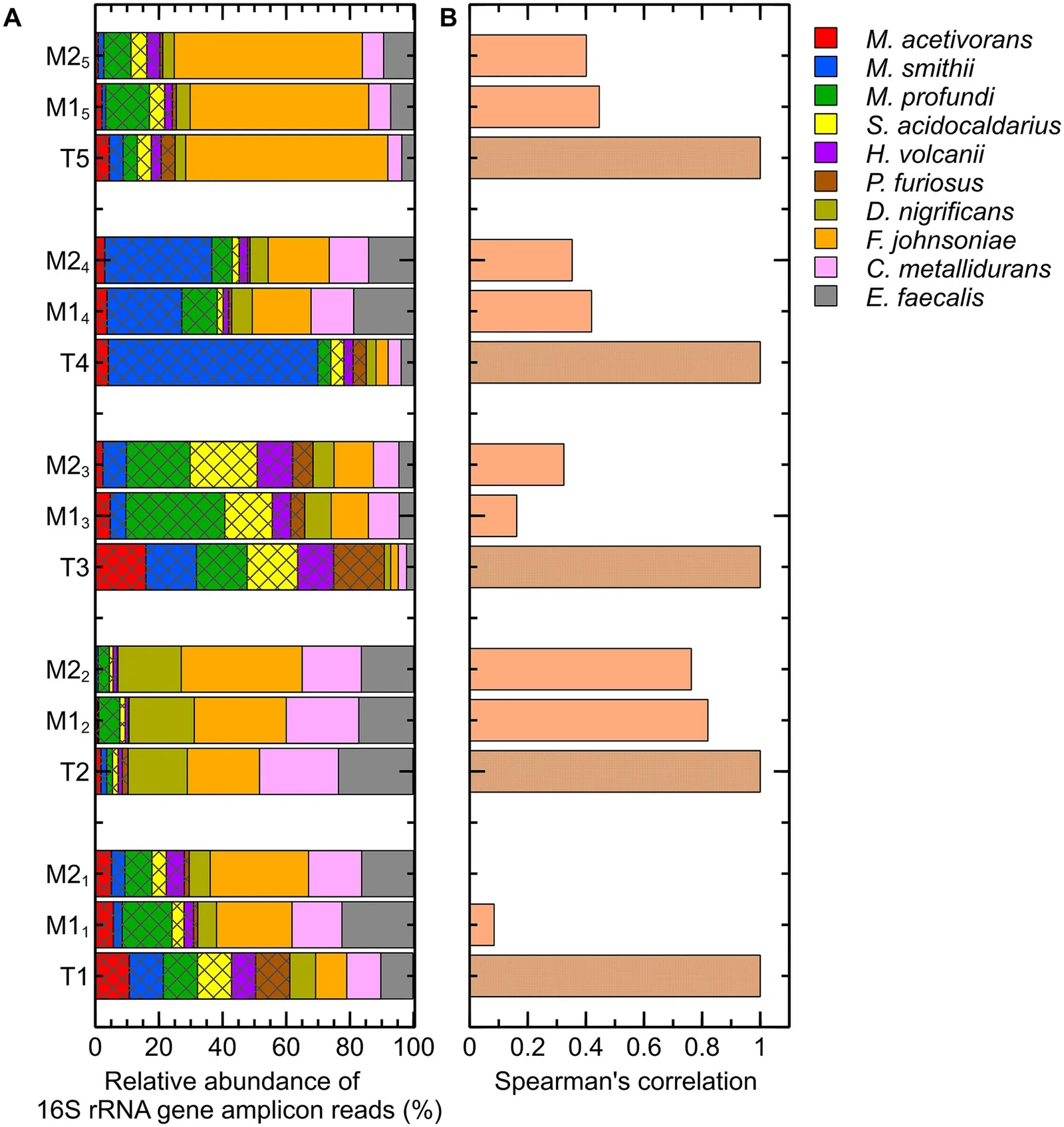
(A) Bar plot visualizing the relative abundances of species present identified with the universal methods M1 and M2 in the different mock samples (M1_x_ or M2_x_) compared to the expected relative abundances of each species (T_x_). Archaea are indicated with a diagonal cross. (B) Bar plot showing the Spearman’s correlation coefficient compared to the theoretical distribution. Spearman’s correlation for M2_1_ versus T1 is −0.0099 and not visible on the graph.

Among the methods intended to be specific for archaea, only M4 and M5 exclusively detected archaeal reads across all mixed mock communities (Fig. 3). All other methods yielded bacterial reads under at least some conditions, although this nonspecific amplification was minimal for M9 and M10 (maximum of 0.03% and only in very few conditions) (Supplementary Fig. 7). Notably, M8 and M9 share the same first primer pair but differ in the second primer pair. Their contrasting performance in mock T2—23% bacterial reads for M8 versus only 0.026% for M9—indicates that the second primer pair substantially influences overall specificity. Method M3 also yielded bacterial reads in all tested conditions (Fig. 3, Supplementary Fig. 7). The results indicate that increasing bacterial abundance is associated with increased nonspecific binding by archaeal primers. This observation is particularly relevant for the analysis of environmental samples, where bacteria are typically expected to dominate over archaea [62–64]. Among the methods that were specific for archaea (M4, M5, M9, and M10), M4 performed best in all conditions based on Spearman correlation coefficients. Although observed associations were only moderate (rho = 0.4), except for T4 where rho = 0.81, the results were consistent for all mock communities, i.e. the over- or underrepresentation of specific taxa did not depend on their relative abundance in the specific mock (Fig. 3). Either if bacteria or archaea were dominant in the mock, the detection of archaea was similar. In fact, this can be generalized for all archaea-specific methods. In addition, all archaea-specific methods had a low specificity for *M. smithii*. For example, for M5, abundances were only 0.46 ± 0.11% in T1, T2, T3, and T5, for M9, relative abundances are 1.61 ± 0.35%, while for M10, only 0.07 ± 0.02% (Supplementary Fig. 7). This probably further explains the lower rho values of these methods.

**Figure 3:**
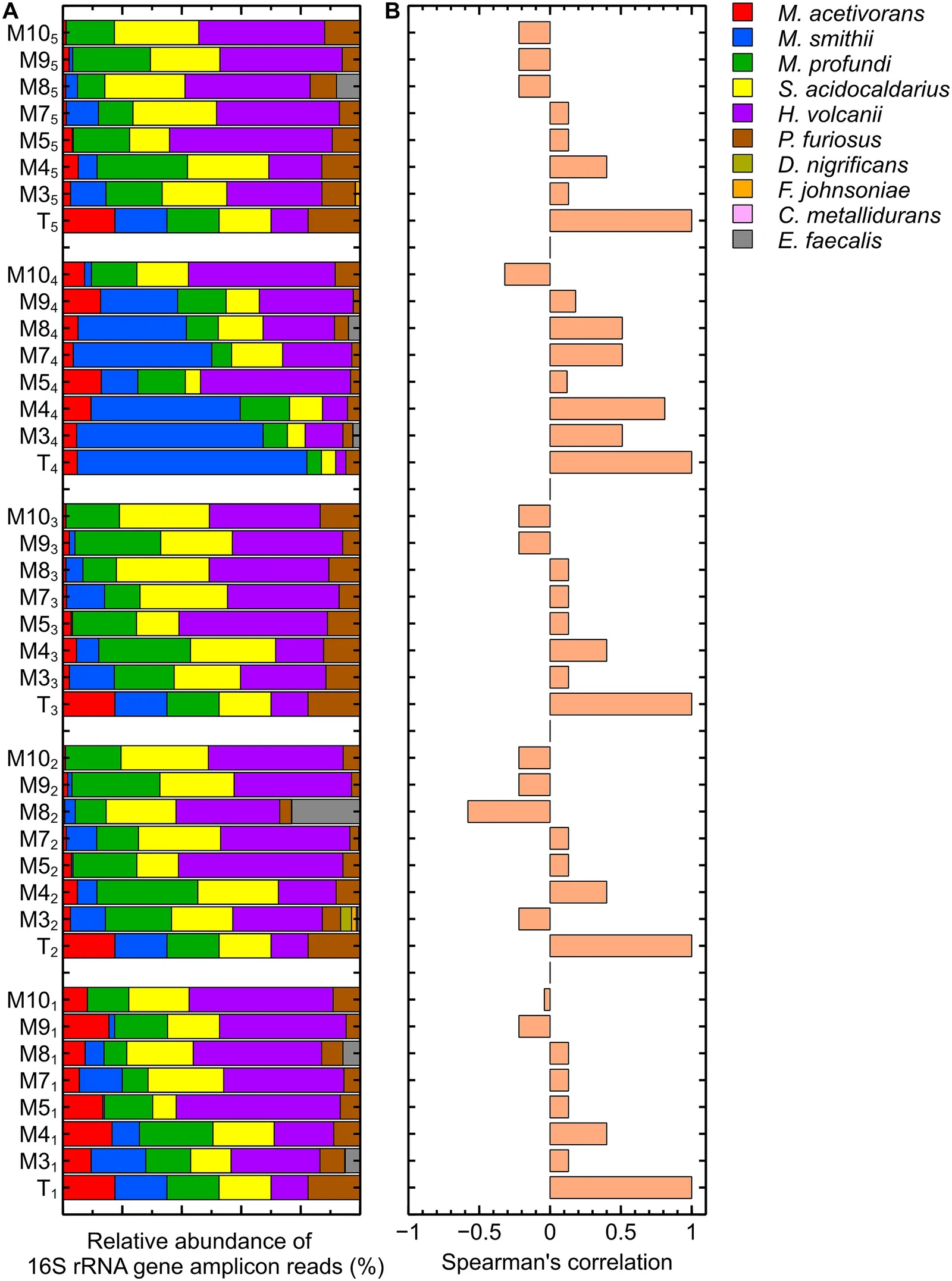
(A) Bar plot visualizing the relative abundances of species present identified with the archaea-specific methods M3–M10 in the different mock samples (Mx_x_) compared to the expected relative abundances of each species (T_x_). (B) Bar plot showing the Spearman’s correlation coefficient compared to the theoretical distribution.

Given the small sample size—where only six archaea should be detected by the archaea-specific methods—and multiple tied values, Spearman’s rank correlation was complemented with ordination to further evaluate the methods (Fig. 4). Similar as for Spearman, compositions identified with most methods yielded similar compositional profiles irrespective of the mock community, except for T1, which were separate. In addition, M3 was more dispersed and this can probably be explained by the differences in bacterial reads that were found in each condition (Fig. 4). Ordination further suggests that M4 approximates the theoretical community composition across the majority of conditions. However, nonspecific amplification is not taken into account in this approach but is instead reflected as a deviation from the expected composition. As nonspecific bacterial amplification is a major disadvantage, we calculated the distance between each method and the theoretical composition while integrating a penalty term for nonspecific amplification (Fig. 5). This underscores the importance of nonspecific amplification in M8 and M3. When methods were ranked within each mock community according to this penalized Aitchison distance score, their rank distributions differed significantly across the five mock communities (Friedman rank-sum test, χ^2^ = 20.66, df = 6, *P*-value = 0.0021; Supplementary Table 4). M4 had the lowest mean within-mock rank (1.2). However, Nemenyi-adjusted post hoc comparisons showed only a significant difference between M4 and M8 (Supplementary Table 4). To assess consistency of M4 performance across mock community conditions, M4 was compared with every other archaea-specific method using exact paired within-mock permutation tests and bootstrap resampling of mock communities. M4 had a lower penalized Aitchison-distance score than M5 and M7–M10 in all five mock communities and a lower score than M3 in four of five mock communities. Descriptive bootstrap 95% intervals for the mean comparator-minus-M4 score differences favored M4 in every comparison, including M3 (0.071–0.565; Supplementary Table 4). Exact permutation tests did not retain statistical significance after Holm adjustment, reflecting the limited number of mock community conditions. Importantly, these results further support M4 as the best method for archaea 16S rRNA gene sequencing. Although there are slight deviations from the theoretical composition, M4 demonstrates low variability across the five mock communities and no detectable nonspecific amplification. Overall, most differences can be explained by nonspecific amplification of bacterial DNA and the lower efficiency in amplifying *M. smithii.*

**Figure 4:**
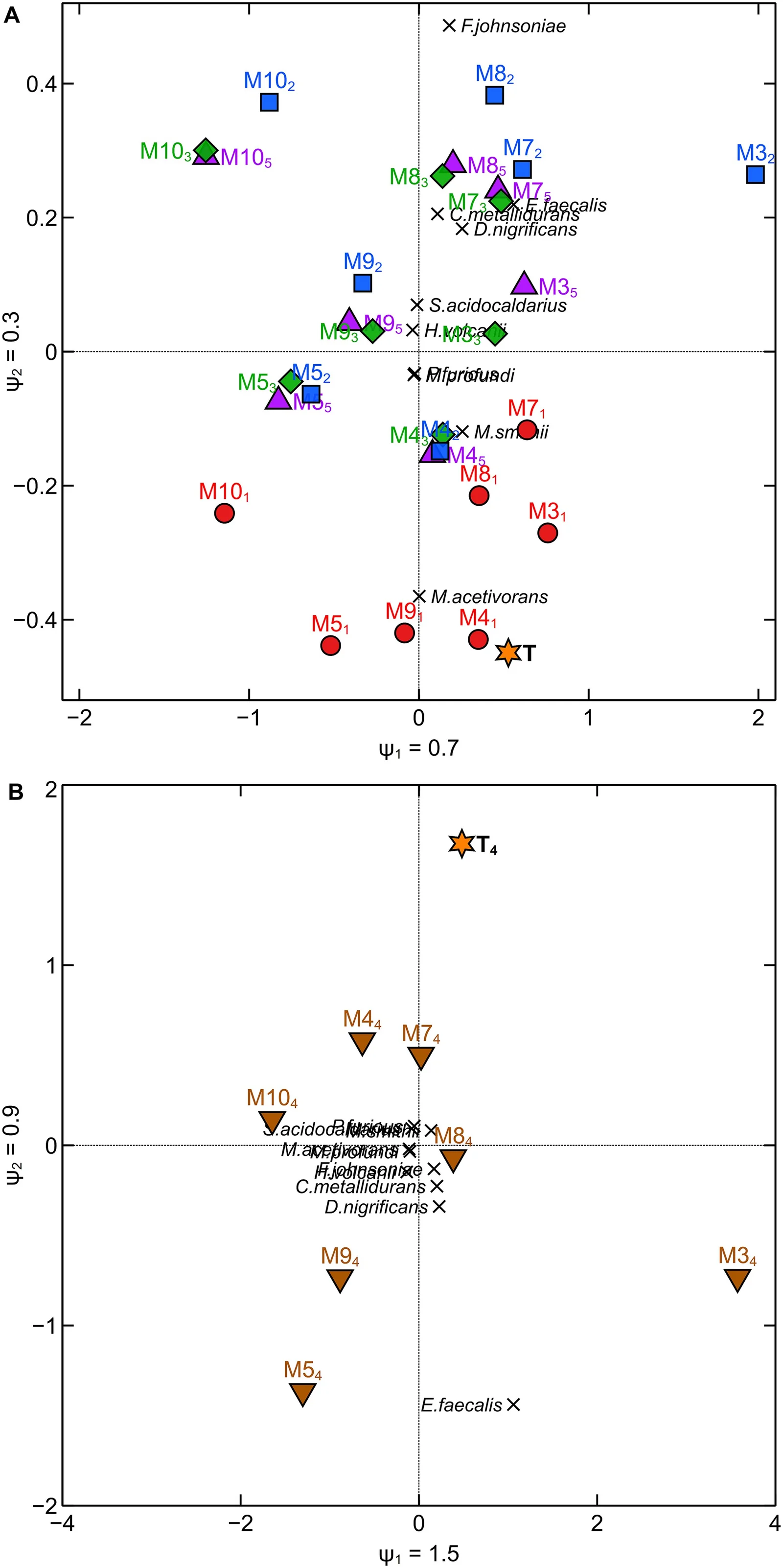
Unconstrained RC(M) biplot visualizing differences in microbial community composition. Plots are split based on different theoretical expected composition. (A) Samples of T1 shown as circles, T2 presented as squares, T3 as triangles, and T5 as diamonds are shown. (B) Samples of T4 presented as inverted triangles are included. The theoretical expected position is shown as star. The species are more abundant in the direction of the x indicated in the graph and less abundant in the opposite direction.

**Figure 5:**
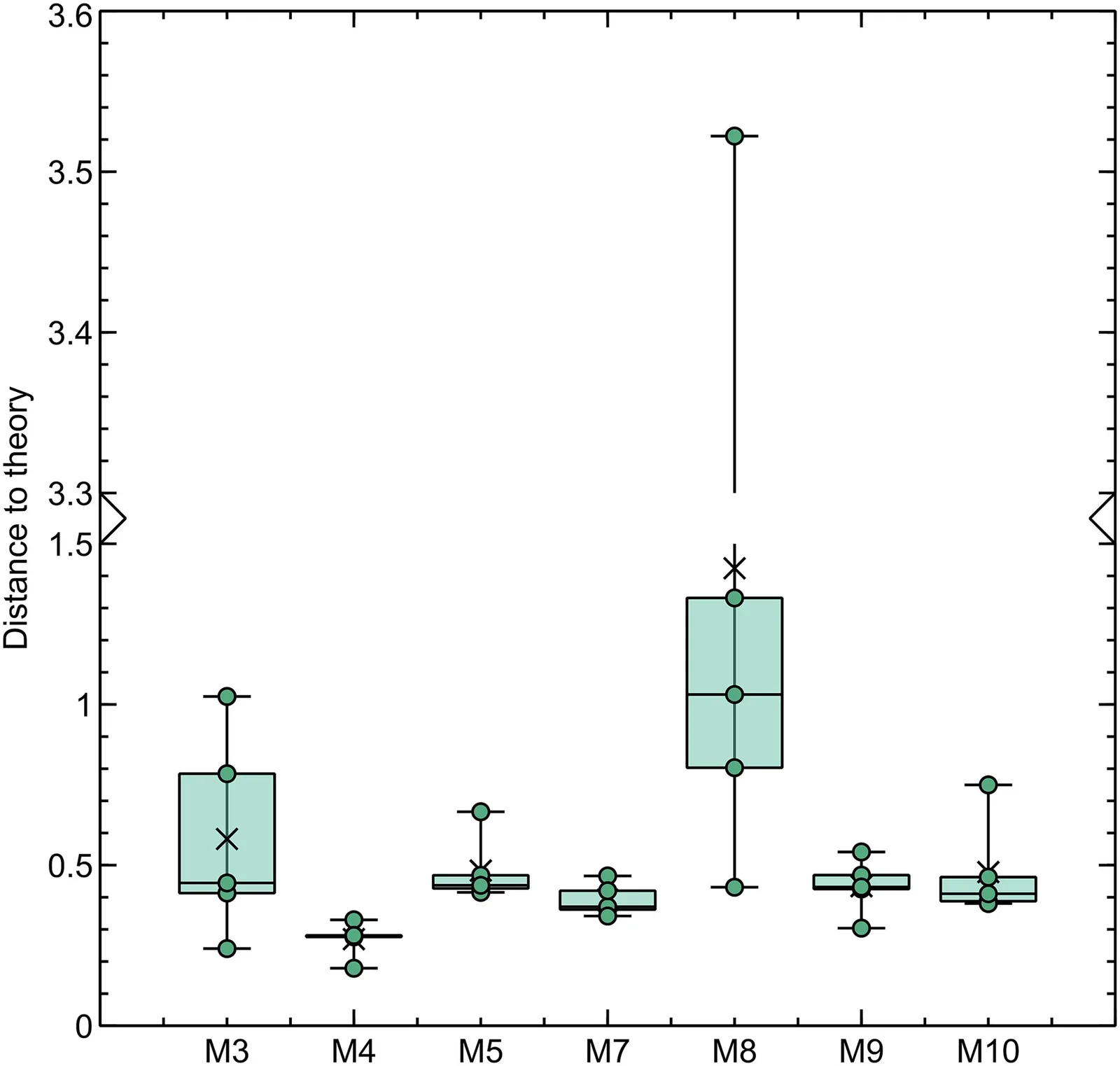
Boxplot showing the distance to theoretical composition across mock community samples. Samples are grouped per primer pair (Mx) and represent one replicate (represented as circle) for each mock community totaling five samples for each primer pair. The box has lines at the lower quartile, median, and upper quartile values. The whiskers are lines extending from each end of the box to show the extent of the rest of the data. The mean read number is indicated with an x. The *y*-axis is broken between 1.5 and 3.3 to accommodate outliers in M8.

#### ASV recovery relative to the expected gene variants in the mock

Several strains in the mock community contain multiple 16S rRNA gene copies, and, for most, these copies are not identical (Table 2). As such, depending on the region of the 16S rRNA gene that is targeted, more than one ASV may be expected for a single strain (Supplementary Fig. 8). Indeed, although *M. smithii* PS carries two different copies, M1, M3, M7, and M8 are expected to result in identical amplicons. Similarly, although *M. acetivorans* C2A contains one divergent copy, all methods used in this study are expected to result in three identical amplicons. Among the bacterial strains, *F. johnsoniae* subsp. *Johnsoniae* UW101 and *E. faecalis* Tissier have multiple copies, with different gene variants, but also here, not more than one ASV is expected with any of the primer pairs. On the other hand, two ASVs are expected for *D. nigrificans* Delft 74.

Overall, the discrepancy between observed and expected ASV for each mock (T1–T5) were small, except for M9 and M10 (Supplementary Fig. 8), and most of the observed differences (e.g. for M3, M7, and M8) can be explained by nonspecific amplification. For M7 and M8, *M. profundi* was represented by four ASV instead of one. The largest discrepancies were observed for M9 and M10, where each archaeal strain was represented by multiple ASV (up to 10 ASVs for *M. profundi*), except *M. smithii.* M9 and M10 are nested PCR methods that share the same nested primer pair, ARC344f–ARC744r. Therefore, the higher ASV counts may reflect the generation of sequence variants with a low abundance during the two-step amplification workflow. Additional PCR amplification and cycle number can increase the occurrence of PCR-derived sequence artifacts, including chimeric sequences and heteroduplex molecules, although the magnitude and type of artifact depend on the specific protocol [11, 60, 65]. Consistent with this interpretation, the nested approaches were indeed more prone to chimera formation (Supplementary Fig. 5).

The two different ASVs of *D. nigrificans* were only identified by M2 and by M3 in mock T2. The two distinct 16S rRNA gene copies of *M. smithii* were not identified by any of the selected methods, and only one ASV was consistently observed. Despite these differences, all methods produced one dominant ASV representing most of the reads for each strain in the mock community. Most taxa were also identified up to the species level, except for *M. profundi* and *P. furiosus,* which could only be identified at the genus level. Nevertheless, blast confirmed 100% identity between the recovered ASVs and the corresponding 16S rRNA gene copies of the strains included in the mock community.

### Technical replication of the best performing archaeal methods

To assess technical variation across repeated experiments, additional replicates of M3 and M4 were generated using exactly the same mock communities and amplification conditions as in the first experiment. We generated two additional replicates for mock T1, T2, and T3 and one additional replicate for mock T4 and T5. Sequencing was performed on a NextSeq 2000 platform instead than the MiSeq platform used for the first series, resulting in an approximately sixfold higher sequencing depth (Supplementary Fig. 9A). Increased sequencing depth increases the sensitivity for low-abundance amplicon variants, which was reflected in the higher number of ASV recovered in the replicates, with 18–23 recovered ASV for M3 and 9–10 for M4, compared to 8–11 for M3 and 6 for M4 in the first experiment (Supplementary Fig. 9B). Notably, although unwanted as it reflects nonspecific bacterial amplification, M3 recovered the two 16S rRNA gene variants of *D. nigrificans* in all samples while it was only observed in mock T2 in the first experiment. Nevertheless, increased sequencing depth may also reveal low-frequency PCR- or sequencing-derived artifacts. The replicate measurements therefore reflect variation across different PCR amplifications and a change in sequencing platform.

Replication confirmed substantial nonspecific bacterial amplification with M3, particularly in mock T2 (48.4% and 65.5% of reads across the two replicates) and T5 (56.1%) where bacterial DNA is present at a higher relative abundance (Supplementary Fig. 10). This nonspecific bacterial amplification was more pronounced in the additional replicates, especially for *F. johnsoniae*, while *P. furiosus* was highly underrepresented in the new replicates. Differences were less pronounced in the mock communities dominated by Archaea (T3 and T4). The observed discrepancies between both experiments cannot be solely explained by differences in sequencing depth. In contrast, M4 generated highly consistent community profiles across the independent PCR runs and sequencing platforms, supporting its robustness for archaeal community profiling.

Penalized Aitchison distance was considered the primary overall performance metric because nonspecific bacterial amplification is a major disadvantage of archaeal targeted 16S rRNA gene sequencing. After accounting for mock community composition, M4 had a significantly lower penalized Aitchison score compared to M3 (F_1,20_ = 15.46, *P*-value = 8.26 × 10^−4^). The estimated M3–M4 difference was 0.684 on the log(1 + score) scale (95% CI [0.321,1.05]), indicating lower integrated compositional error and bacterial co-amplification for M4. This result was further supported by a restricted within-mock permutation test (P_perm_ = 1 × 10^−4^, 9999 permutations). As the first and second series of samples were sequenced on different platforms, not all variability can be attributed solely to PCR amplification. Nevertheless, differences between M4 and M3 remained significant if the sequencing platform was included as an additional covariate (*P*-value = 2.93 × 10^−4^) and if it was restricted to the additionally generated replicates (*P*-value = 4.62 × 10^−4^). Altogether, these results underline the suitability of M4 for 16S rRNA gene sequencing of archaea, both in terms of specificity and reproducibility across technical and platform-level variation.

### Validation of primer pairs on environmental samples

Boom Clay pore water has been shown to harbor an active methanogenic microbial community; however, the precise composition of the archaeal community is not yet known [66, 67]. To elucidate this, Boom Clay pore water samples were previously subjected to 16S rRNA gene sequencing with a standard commercial approach using the primer pair U519F–Ar9R, comparable to M3 used in the present study. However, the PCR conditions could be different as these were not reported by the company. These results indicated that the method was not specific for archaea as the proportion of bacterial reads ranged from 2.4% to 99.9% across the samples with an average of 53.6% ± 37.7% (Supplementary Fig. 11). Despite possible differences in PCR conditions, we also observed nonspecific bacterial amplification with M3 ranging from 1.7 ± 1.5% in the mock T3 with containing the highest relative abundance of archaea to 40.1 ± 30.3% in mock T2 containing the lowest amount of archaea (Fig. 3, Supplementary Figs 7 and 10). This suggests that the lack of specificity of M3 is inherent to the primer pair and is not suitable to monitor archaea in environmental samples. Based on our result with the mock communities, we used M4 to reanalyze two Boom Clay pore water samples for which residual DNA was available for an additional analysis (Fig. 6). No nonspecific amplification was observed, further confirming the selectivity of this method towards Archaea. In addition, only few archaeal genera could be detected with M3, i.e. *Methanolobus* and unclassified *Bathyarchaeia* (but abundance only 0.05%) in Sample 17 and the species *Methanobacterium _D sp963785285* in Sample 15. In contrast, reanalysis with M4 showed a higher archaeal diversity in the Boom Clay pore water samples with a variety of methanogenic archaea (Fig. 6). Archaeal composition differed between the two samples, with *Methanobacterium* predominating in Sample 15 and *Methanolobus* detected prominently in Sample 17. Both samples were included solely to demonstrate the effect of primer choice on archaeal detection. The present dataset is too limited to support an ecological interpretation or to attribute this difference to specific environmental factors.

**Figure 6:**
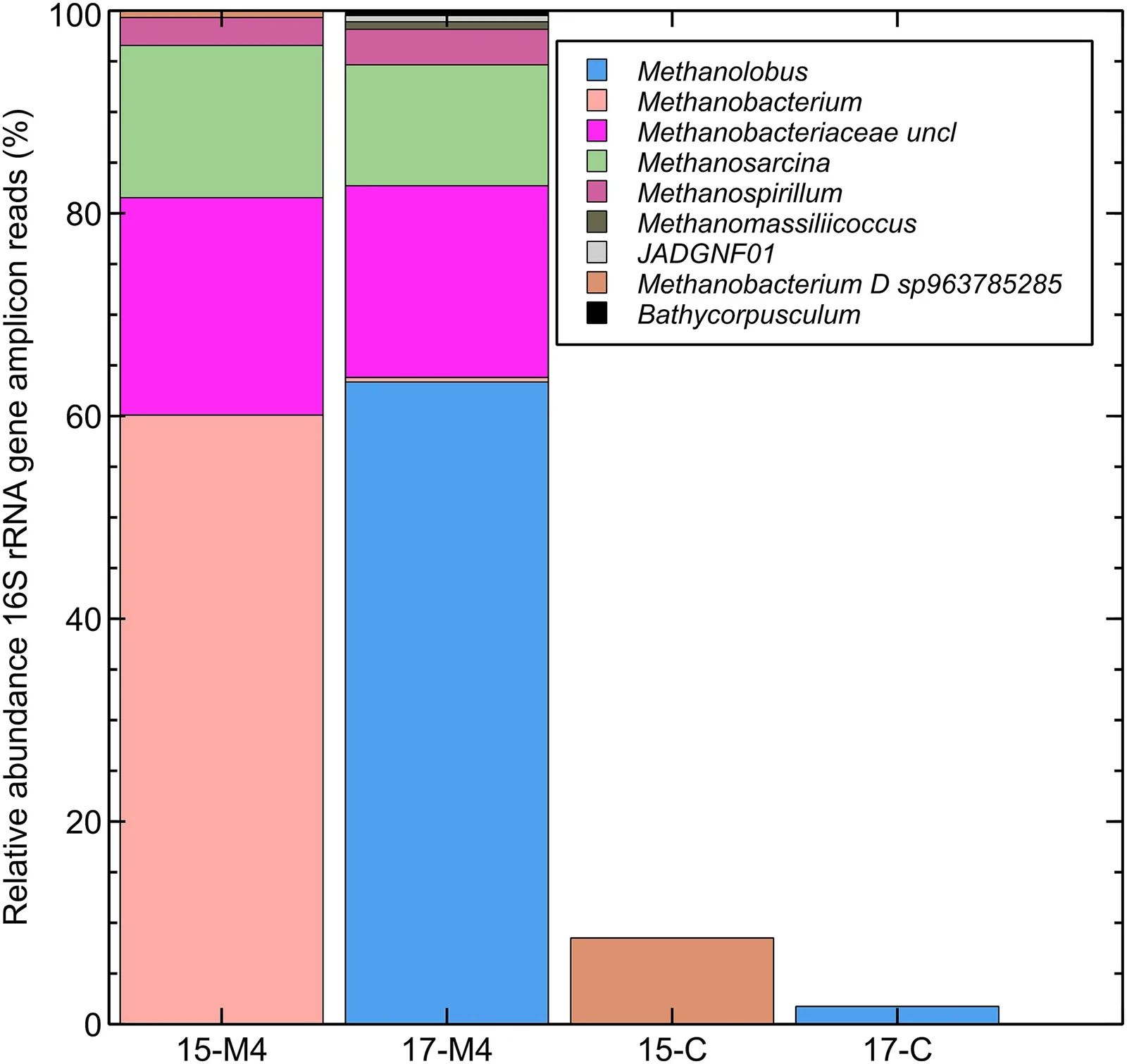
Bar plot showing the relative abundances of 16S rRNA gene sequencing reads from different Archaea obtained after applying M4 on Sample 15 and 17 (15-M4 and 17-M4) of Boom Clay pore water compared to the relative abundances obtained after the standard commercial procedure (15-C and 17-C).

Finally, to further confirm the specificity of M4, it was used to amplify in duplicate HC227, a complex bacterial mock community consisting of DNA from 227 strains [49]. Following the same DADA2 workflow as for our mock communities, we detected 23 and 25 non-chimera sequences in both replicates, representing only 0.02% of the input reads. The obtained ASVs were identified as Archaea (two ASVs) indicating that they likely represent artefactual sequences (Supplementary Table 5). These results further validated the high selectivity of M4 for Archaea.

In conclusion, this study evaluated two universal and eight archaeal-specific 16S rRNA gene primer pairs on a newly developed mock community comprised of bacteria and archaea, with a focus on methanogenic archaea. Our data indicate that even with a relatively simple mock community, the universal methods tested here are generally not recommended for accurate simultaneous quantification of bacterial and archaeal relative abundances as they consistently overestimated bacterial abundances. Among the archaea-specific primer pairs tested, M4 (Arch349F 5′-GYGCASCAGKCGMGAAW-3′ – A806R 5′-GGACTACVSGGGTATCTAAT-3′) proved most suitable to detect archaea in 16S rRNA amplicon sequencing studies, even when bacteria are highly abundant. Reanalysis of Boom Clay pore water samples further confirmed that M4 detected archaeal diversity that was missed or severely underestimated by the previously used commercial method (M3). Overall, this work demonstrates that benchmarking through mock communities is essential to evaluate archaeal primer performance. Further validation with archaeal mock communities covering a broader phylogenetic range can give additional proof of the suitability of M4.

## Supplementary Material

Supplementary_material_ycag295

## Data Availability

The sequence datasets generated and analyzed during this study are available in the NCBI SRA repository (PRJNA1444866).
