# Supplementary material for: Improved detection of methanogenic archaea by validating 16S rRNA gene primers using defined archaeal–bacterial mock communities
Source: ISME Commun. 2026 Oct 13;6(1):ycag295. doi: 10.1093/ismeco/ycag295 (PMC13747024; doi:10.1093/ismeco/ycag295)
Supplement: Supplementary file 1 [file supplementary_material_ycag295.zip › Supplementary_Information_ycag295.docx]

**Supplementary Information**

**Improved detection of methanogenic archaea by validating 16S rRNA gene primers using defined archaeal–bacterial mock communities**

**Mijnendonckx Kristel^*1^, Provoost Ann^1^, Smolders Carla^1^, Leys Natalie^1^**

^1^Microbial Biotechnology Unit, Belgian Nuclear Research Centre, SCK CEN, Mol, Belgium

***Correspondence:**
Kristel Mijnendonckx

Microbial Biotechnology Unit

Belgian Nuclear Research Centre, SCK CEN

Boeretang 190

2400 Mol

Belgium

kristel.mijnendonckx@sckcen.be

Supplementary Figure 1: Relative abundance of ASV belonging to *Flavobacterium* (A) or *Desulfomaculaceae* (B) after processing the 16S rRNA gene sequencing data of 17 Boom Clay porewater samples with a standard commercial approach for Archaea (red bars) and Bacteria (blue bars). Only samples that were positive in either condition are shown.

Supplementary Figure 2: Schematic representation of the HADES URF in Boom Clay (Mol Belgium). Figure based on [1].

[1.] De Craen, M., H. Moors, and J. Verstricht, Description of the HADES piezometers used for the study of in situ Boom Clay pore water chemistry. SCK•CEN Reports. 2019: Studiecentrum voor Kernenergie.

Supplementary Figure 3: Heatmap visualizing the coverage of each method across archaeal phyla based on *in silico* analysis of each primer pair. Only methods for which coverages were estimated with Silva TestPrime 1.0 are shown. For the nested PCR approaches (M7-M10), results of the first primer pair evaluated with Silva TestPrime 1.0 are shown.

Supplementary Figure 4: Boxplots showing the total number of reads in the samples. Samples are grouped per primer pair (Mx) and represented by one replicate (represented by a diamond) for each mixed mock community (T1-T5) (totaling 5 data points for each primer pair Mx). The box has lines at the lower quartile, median and upper quartile values. The whiskers are lines extending from each end of the box to show the extent of the rest of the data. The mean read number is indicated with an x.

Supplementary Figure 5: Boxplots showing the percentage of reads retained after filtering (blue), merging (green) and chimera removal (red) in the samples. Samples are grouped per primer pair (Mx) and represent one replicate (represented by a diamond) for each mixed mock community (totaling 5 for each primer pair). The box has lines at the lower quartile, median and upper quartile values. The whiskers are lines extending from each end of the box to show the extent of the rest of the data. The mean read number is indicated with an x.

Supplementary Figure 6: Overview of the relative abundance of each of the ten strains present in the five different mock composition samples (T1-T5) as identified with the universal primer pairs used in methods M1 and M2. Theoretical abundances are indicated with T_x_ and bars are filled with dots.

Supplementary Figure 7: Overview of the relative abundances of each of the ten strains present in the five different mock communities (T1-T5) identified with the archaea-specific primer pairs used in methods M3-M10. Theoretical abundances are indicated with T_x_ and bars are filled with dots.

Supplementary Figure 8: Boxplots showing the total number of identified ASV with a least two reads for each method compared to the expected amount of ASV for each method (orange cross). Samples are grouped per primer pair (Mx) and represent one replicate (represented by a circle) for each mixed mock community (totaling 5 for each primer pair). The box has lines at the lower quartile, median and upper quartile values. The whiskers are lines extending from each end of the box to show the extent of the rest of the data. The mean ASV number is indicated with an x.

Supplementary Figure 9: Boxplots showing the total number of A) reads and B) ASV for M3 and M4 obtained after sequencing on the Miseq platform (indicated with a) or on the NextSeq 2000 platform (indicated with b). Samples are grouped per primer pair. The box has lines at the lower quartile, median and upper quartile values. The whiskers are lines extending from each end of the box to show the extent of the rest of the data. The mean read number is indicated with an x.

Supplementary Figure 10: Bar plot visualizing the relative abundances of species present identified with the archaea-specific methods M3 and M4 in the different mock samples (Mx_x_) compared to the expected relative abundances of each species (T_x_). Archaea are indicated with a diagonal cross. Different replicates are indicated with numbers.

Supplementary Figure 11: Relative abundance of bacterial (blue) and archaeal reads (red) detected after processing 16S rRNA gene sequencing data of 17 Boom Clay pore water samples with a standard commercial approach.

Supplementary Table 1: Classification of the microbial strains present in the mock community.

| Kingdom | Phylum | Class | Order | Family | Genus | Species | Strain |
| --- | --- | --- | --- | --- | --- | --- | --- |
| Archaea | Halobacteriota | Methanosarcinia | Methanosarcinales | Methanosarcinaceae | *Methanosarcina* | *acetivorans* | C2A |
| Archaea | Methanobacteriota | Methanobacteria | Methanobacteriales | Methanobacteriaceae | *Methanobrevibacter* | *smithii* | PS |
| Archaea | Halobacteriota | Methanosarcinia | Methanosarcinales | Methanosarcinaceae | *Methanolobus* | *profundi* | MobM |
| Archaea | Thermoproteota | Thermoproteia | Sulfolobales | Sulfolobaceae | *Sulfolobus* | *acidocaldarius* | 98-3 |
| Archaea | Halobacteriota | Halobacteria | Halobacterales | Haloferacaceae | *Haloferax* | *volcanii* | DS2 |
| Archaea | Methanobacteriota | Thermococci | Thermococcales | Thermococcaceae | *Pyrococcus* | *furiosus* | Vc1 |
| Bacteria | Bacillota | Desulfotomaculia | Desulfotomaculales | Desulfotomaculaceae | *Desulfotomaculum* | *nigrificans* | Delft 74 |
| Bacteria | Bacteroidota | Bacteroidia | Flavobacteriales | Flavobacteriaceae | *Flavobacterium* | *Johnsoniae* subsp. *Johnsoniae* | UW101 |
| Bacteria | Pseudomonadota | Gammaproteobacteria | Burkholderiales | Burkholderiaceae | *Cupriavidus* | *metallidurans* | CH34 |
| Bacteria | Bacillota | Bacilli | Lactobacillales | Enterococcaceae | *Enterococcus* | *faecalis* | Tissier |

Supplementary Table 2: Overview of the parameters used for filtering and trimming the 16s rRNA gene sequencing reads in each method. Other parameters were kept at their default settings.

| Method | Primer name | Sequence 5’-3’ | TruncLen (Fw, Rv) | maxEE (Fw, Rv) |
| --- | --- | --- | --- | --- |
| M1 | 807F  1050R | GGATTAGATACCCBRGTAGTC  AGYTGDCGACRRCCRTGCA | 220, 180 | 2, 2 |
| M2 | Pro341F  Pro805R | CCTACGGGNBGCASCAG  GACTACNVGGGTATCTAATCC | 280, 200 | 2, 4 |
| M3 | U519F  Ar9R | CAGCMGCCGCGGTAA  CCCGCCAATTCCTTTAAGTTTC | 250, 160 | 2, 4 |
| M4 | Arch349F  A806R | GYGCASCAGKCGMGAAW  GGACTACVSGGGTATCTAAT | 250, 160 | 2, 4 |
| M5 | SSU1bF*  SSU520R | CCGGTTGATCCWGSCRBA  GCTACGRRYGYTTTARRC | 280, 240 | 2, 4 |
| M7-M8 | U519F  A806R | CAGCMGCCGCGGTAA  GGACTACVSGGGTATCTAAT | 180, 130 | 1, 1 |
| M9-M10 | ARC344f  ARC744r | ACGGGGCGCAGCAGGCGCGA  CCSGGGTATCTAATCC | 260, 190 | 2 ,4 |

Supplementary Table 4: Rank based and targeted comparison of M4 with other archaea-specific primer pairs across the five mock-community compositions. significant p-values are indicated in bold. n.a.: not applicable

| Method/  Comparator | Mean within-mock rank | Rank 1 | Nemenyi adjusted p-value, M4 versus method | Mean paired difference in penalized Aitchison score, comparator - M4 | Mock communities  favoring M4 | Exact two-sided  permutation  p-value | Holm adjusted  p-value | Bootstrap 95% CI |
| --- | --- | --- | --- | --- | --- | --- | --- | --- |
| M4 | 1.2 | 4/5 | n.a. | n.a. | n.a. | n.a. | n.a. | n.a. |
| M3 | 4.4 | 1/5 | 0.22 | 0.31 | 4/5 (not T3) | 0.1250 | 0.375 | 0.07 - 0.57 |
| M5 | 4.8 | 0/5 | 0.12 | 0.21 | 5/5 | 0.0625 | 0.375 | 0.14 – 0.31 |
| M7 | 2.8 | 0/5 | 0.91 | 0.12 | 5/5 | 0.0625 | 0.375 | 0.08 – 0.16 |
| M8 | 7 | 0/5 | **0.00044** | 1.15 | 5/5 | 0.0625 | 0.375 | 0.41 - 2.23 |
| M9 | 4.2 | 0/5 | 0.30 | 0.16 | 5/5 | 0.0625 | 0.375 | 0.13 – 0.22 |
| M10 | 3.6 | 0/5 | 0.57 | 0.21 | 5/5 | 0.0625 | 0.375 | 0.12 – 0.35 |

Supplementary Table 5: Overview of amplicon sequence variants (ASV), their classification and the amount of reads obtained after analysis of 16S rRNA gene sequencing data obtained after applying method 4 (M4) on the mock community HC227. Analysis was performed in duplicates indicated by _1 and _2.

| ASV | Kingdom | Phylum | Class | Order | Family | Genus | HC227_1 | HC227_2 |
| --- | --- | --- | --- | --- | --- | --- | --- | --- |
| ASV1 | Archaea | *Halobacteriota* | *Methanosarcinia* | *Methanosarcinales* | *Methanosarcinaceae* | *Methanosarcina* | 23 | 6 |
| ASV2 | Archaea | *Methanobacteriota* | *Methanobacteria* | *Methanobacteriales* | *Methanobacteriaceae* | *Methanobacterium* | 0 | 19 |
